# Temperature and precipitation effects on the isotopic composition of global precipitation reveal long-term climate dynamics

**DOI:** 10.1038/s41598-021-98094-6

**Published:** 2021-09-16

**Authors:** Y. Vystavna, I. Matiatos, L. I. Wassenaar

**Affiliations:** Isotope Hydrology Section, Vienna International Centre, International Atomic Energy Agency, PO Box 100, 1400 Vienna, Austria

**Keywords:** Hydrology, Atmospheric dynamics

## Abstract

Earth’s climate history is traced through the long-term covariance between the isotopic (*δ* ^18^O) composition of archived meteoric waters (groundwater, ice cores) with air temperature (T) and amount of precipitation (P). To assess recent multi-decadal climatic changes, we analysed *δ*^18^O, T and P, and the relationships between these parameters at 20 stations having 60 years of continuous monthly isotopic records. Using nonparametric regressions and time series modelling we found significant linear and non-linear relationships for *δ*^18^O with T and P and showed that the *δ*^18^O dependency on these two parameters varied over decadal scales, thereby revealing complex relationships related to recycled moisture, large-scale convective processes and atmospheric-oceanic oscillations. Due to multiple factors controlling the *δ* ^18^O composition of precipitation including P and T effects, we found that time-varying relationships between *δ*^18^O in precipitation P and T were better explained using the non-linear regressions. Our results affirmed that *δ*^18^O distributions in global precipitation are integrative indicators of climate dynamics whose patterns can be applied to better understand region-specific climatic changes in the present, past, and future.

## Introduction

It is well-established that the stable isotopic (*δ*^18^O and *δ*^2^H) composition of precipitation is primarily dependent on two key climatic variables: air temperature (T) and precipitation amount (P) which together vary seasonally, annually and spatially^1–3^. The strong relationship between the *δ*^18^O composition of meteoric water and T have long been used to map Earth’s climatic and temperature history via precipitation-driven proxy isotope records like *δ*^18^O in ice cores, cellulose in tree rings, freshwater shells, carbonates in lake cores and cave speleothems, thereby allowing detailed reconstruction of Earth’s climate and temperature over millennial timescales^4–9^. For most climatic isotopic proxies, *δ*^18^O trends in water and proxies are generally interpreted and modelled as time-averaged *linear* responses to T and/or P at stationary locations (i.e., ice cores) over geologic time^7,8,10–16^. Bowen^17^, however, noted that at some meteorological stations the relationships between *δ*^18^O in contemporary precipitation to T, P and other meteorological parameters was highly non-linear when evaluated by nonparametric statistics. Non-linear relationships between *δ*^18^O and various meteorological parameters were attributed to complex atmospheric processes acting over various spatial domains and timescales^17–20^. However, the T and P effects on the *δ*^18^O composition of precipitation are treated by most researchers as linear constants over the long-term^12,16,21^.

To better understand how T and P control the temporal variability of *δ*^18^O in precipitation (and by extension climate proxies) and which of these covariates has the largest influence on the climatic isotopic signal at different locations over time, we evaluated the relationships between *δ*^18^O in precipitation with T and P at 20 global meteorological stations having long-term monthly *δ*^18^O and climate records spanning ca. 60 years. Our hypothesis was that the dependency of *δ*^18^O on T and/or P is time-varying due to inter-related local and large-scale hydroclimate processes that alter the linear trends of the *δ*^18^O–T and -P relationships. These findings provide insights into the integrative nature of *δ*^18^O in precipitation that need to be more deeply considered in the interpretation of oxygen isotope variations in climate proxies.

## Results and discussion

### Nonparametric T and P effects at long-term stations

**Figure 1:**
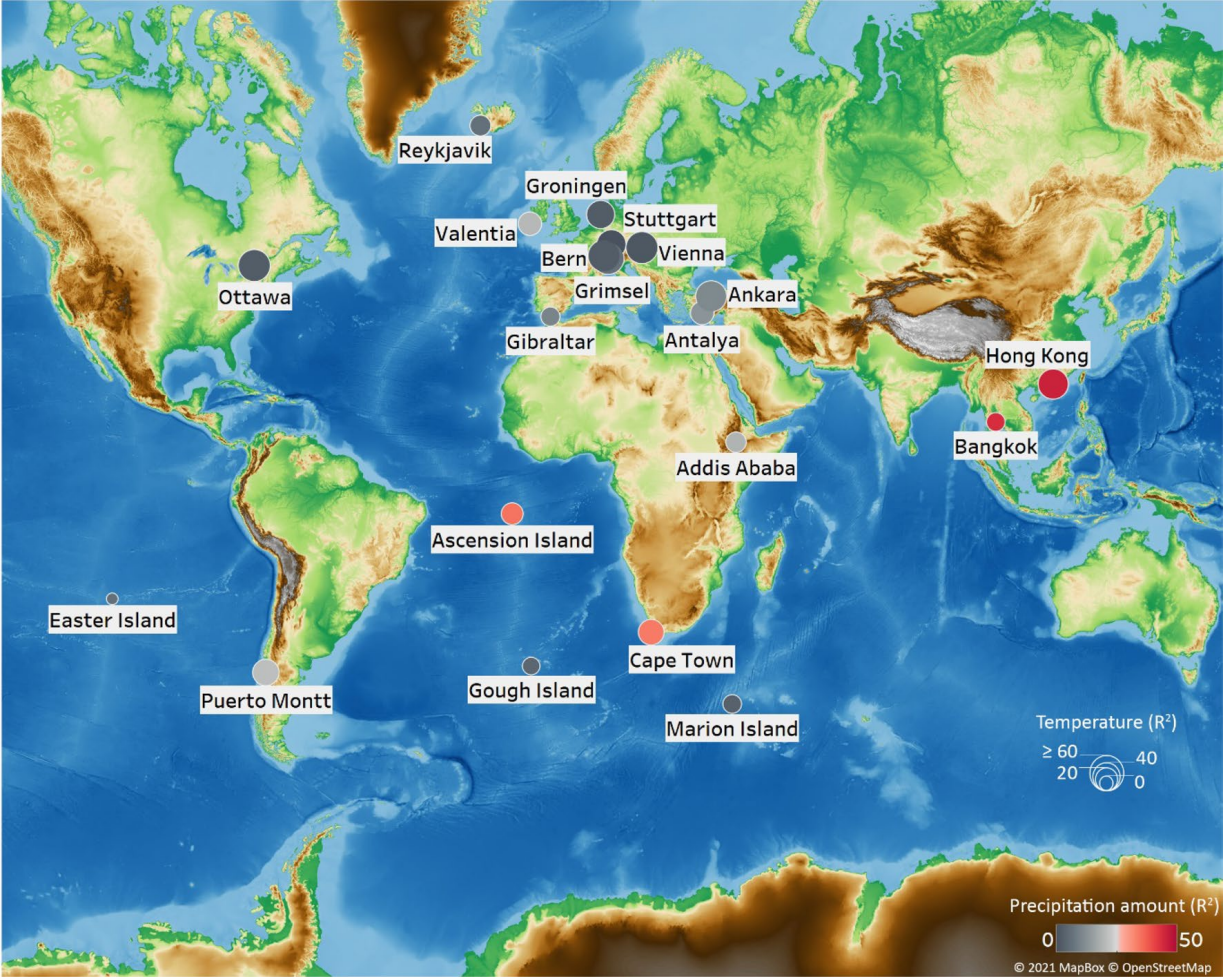
The location of long-term GNIP stations and the coefficient of determination (R^2^) of *δ*^18^O–T and *δ*^18^O–P nonparametric relationships. Base map is from OpenStreetMap and OpenStreetMap Foundation under the Open Database License (https://www.openstreetmap.org/copyright).

Based on nonparametric and linear relationships between *δ*^18^O, T and P, our stations (Fig. 1) were categorized into four groupings (Table SI-1) or classic patterns previously recognized^1,22^: (Group 1) where *δ*^18^O was mainly controlled by T (continental stations: Ankara, Bern, Grimsel, Groningen, Stuttgart, Ottawa and Vienna); (Group 2) where *δ*^18^O was mainly controlled by P (Ascension Island and Bangkok); (Group 3) where *δ*^18^O was equally controlled by T and P (Addis Ababa, Antalya, Cape Town, Hong Kong, Puerto Montt and Valentia); and (Group 4) where *δ*^18^O was poorly explained by T or P (Easter Island, Gibraltar, Gough Island, Marion Island and Reykjavik).

**Figure 2:**
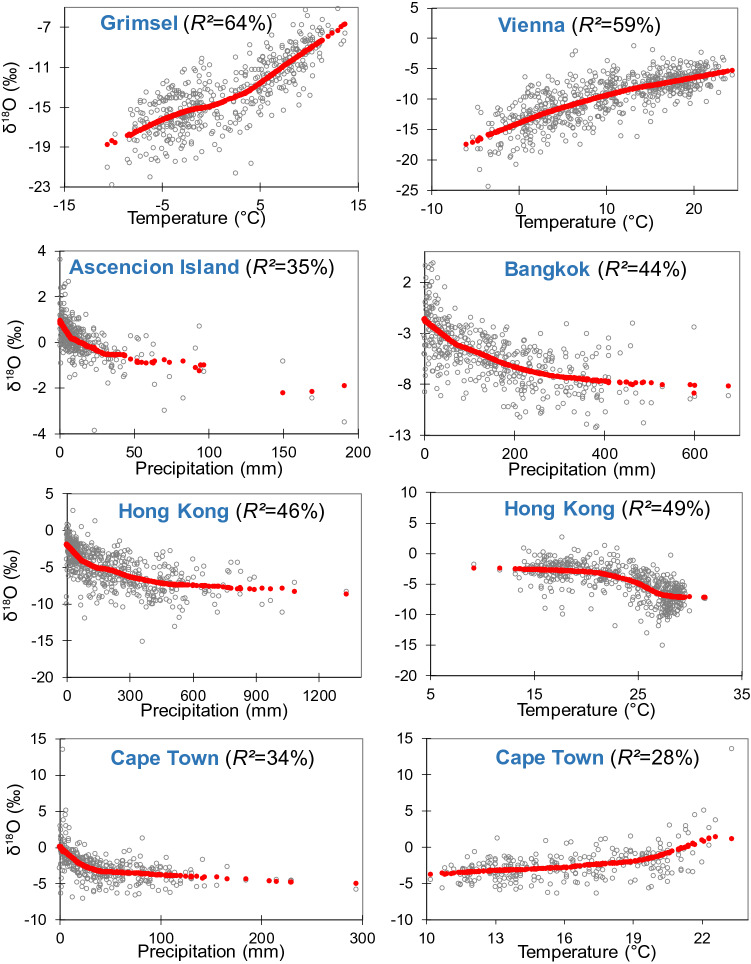
Polynomial trends (red) fitted by nonparametric regression models for *δ*^18^O versus T and P (grey dots) for selected long-term GNIP stations.

For Group 1 significant T effects (by *R*^2^) varied from 39% in Groningen to 70% in Ottawa (Fig. 1, Table SI-1) that can be explained by different proportions of admixing of oceanic air masses transported to the continent with recycled moisture from the land surface (transpiration, evaporation of the soil water and surface water)^21,23,24^. The contribution of recycled moisture is magnified when air temperatures rises above a threshold^23–25^, which explains the non-linear *δ*^18^O–T relationship in this group (Fig. 2). Having moisture origins from isotopically depleted water sources, the admixture of recycled moisture influences the *δ*^18^O–T trend mainly during the warm season when T is ˃10–15 °C (Fig. 2, Figure SI-1). For Group 2 (Ascension Island, Bangkok, Cape Town, and Hong Kong) the nonparametric regressions had higher significance based on their coefficients of determination than linear regressions (*R*^2^) (Table SI-1).

A decreasing trend in the *δ*^18^O–T relationship revealed a lower T effect in Ankara, Ottawa and Vienna for the summer warm period (May–October) due to more intensive evaporation and transpiration. The *δ*^18^O–T relationship for these stations was stronger during the cold season (November–April) (Table 1). By contrast, Bern, Grimsel and Stuttgart had stronger *δ*^18^O–T effects during the warm period but were lower in the cold season (November–April) (Table 1). The higher importance of the T effect in Grimsel and Bern can be explained by the fact that summer temperatures at these stations are lower than Ottawa and Vienna, and that the T effect threshold (10–15 °C) for significant contributions from recycled moisture are not fully reached (Fig. 2 and Figure SI-1). Grimsel displayed the strongest *δ*^18^O–P relationship (*R*^2^ = 17%) among the stations in this grouping. Most stations (except Ottawa and Vienna) had higher P effects during the warm period, suggesting that *δ*^18^O is partly controlled by larger scale convective processes in the summer season (Table 1).

**Table 1:** Coefficients of determination (*R*^2^, %) between *δ*^18^O, T and P in May–October (warm period) and November–April (cold period).

Station	*R*^2^ in May–October, %		*R*^2^ in November–April, %	
	*δ*^18^O–T	*δ*^18^O–P	*δ*^18^O–T	*δ*^18^O–P
Ankara	22	9	32	1
Bern	46	3	18	0
Grimsel	53	17	14	0
Groningen	12	14	17	3
Stuttgart	27	4	13	2
Ottawa	22	1	53	3
Vienna	25	4	37	4

The *δ*^18^O–P correlation for Ascension Island and Bangkok (Group 2) was much stronger than the stations in Group 1, and more significant than the *δ*^18^O–T relationship (Fig. 1, Table SI-1). Beyond a threshold in P (< 100 mm for Ascension Island and < 400 mm in Bangkok), the trend of the *δ*^18^O–P relationship declined and became constant (Fig. 2). Both stations receive precipitation from Pacific oceanic moisture. For Ascension Island, with an oceanic arid climate having extremely low and sporadic precipitation^1^, large convective processes from Pacific Ocean episodically^26^ bring high amounts of isotopically depleted precipitation^13,27,28^. In Bangkok, heavy rains originate from Pacific moisture having a lower ^18^O isotopic composition compared to recycled moisture and other sources^29,30^, which explains the lack of a strong T effect.

Bangkok (Group 2) and Hong Kong (Group 3) are also influenced by oceanic convective processes^29,31,32^, and thereby have a strong P effect explaining ca. 45% of the *δ*^18^O variance, concomitant with a decrease in the *δ*^18^O–T relationship (*R*^2^ = 50%). Non-linear modelling showed a decreasing *δ*^18^O–T trend from low T to 20 °C, a sharply decreasing trend at 27 °C, and a constant *δ*^18^O trend at higher T (Fig. 2). This contrasted with the *δ*^18^O–T relationship for other stations with strong T effects (Fig. 2), and a clear T threshold suggests a partial role of rising temperature on *δ*^18^O particularly at T > 20 °C^19^. Enhanced evaporation of oceanic water provides local water vapor more enriched in ^18^O mainly during non-monsoonal season^33^.

For Addis Ababa, Puerto Montt, Cape Town and Valentia (Group 3) the variations in *δ*^18^O were equally explained by T and P (Fig. 1). Cape Town showed a positive *δ*^18^O–T relationship up to ca. 18 °C, but the trend increased rapidly beyond this T threshold, suggesting the intensification of the T effect and less contribution of recycled moisture compared to stations in Group 1 with a dominant T effect (Figs. 1 and 2, Table SI-1). Decreased contribution of recycled moisture and intensification of temperature effects under T growth was also observed for Addis Ababa, Antalya, Puerto Montt and Valentia. However, Cape Town, Valentia, Antalya and Puerto Montt had a negative correlation for *δ*^18^O–P with a rapidly decreasing trend to a threshold, suggesting that higher P originates from larger scale convective processes (Fig. 2, Figure SI-2). Decreasing P, rising *δ*^18^O and T indicate a link between *δ*^18^O and T over time due to the intensification of the sub-cloud evaporation^19,30,34^. The stations on the oceanic islands (Group 4) revealed weak *δ*^18^O correlations with T or P (Fig. 1) confirming negligible land mass influences^1^ and a strong and direct impact of oceanic moisture on the isotopic composition for these islands^17,19,35^.

### Long-term dependency of δ^18^O on T and P

**Figure 3:**
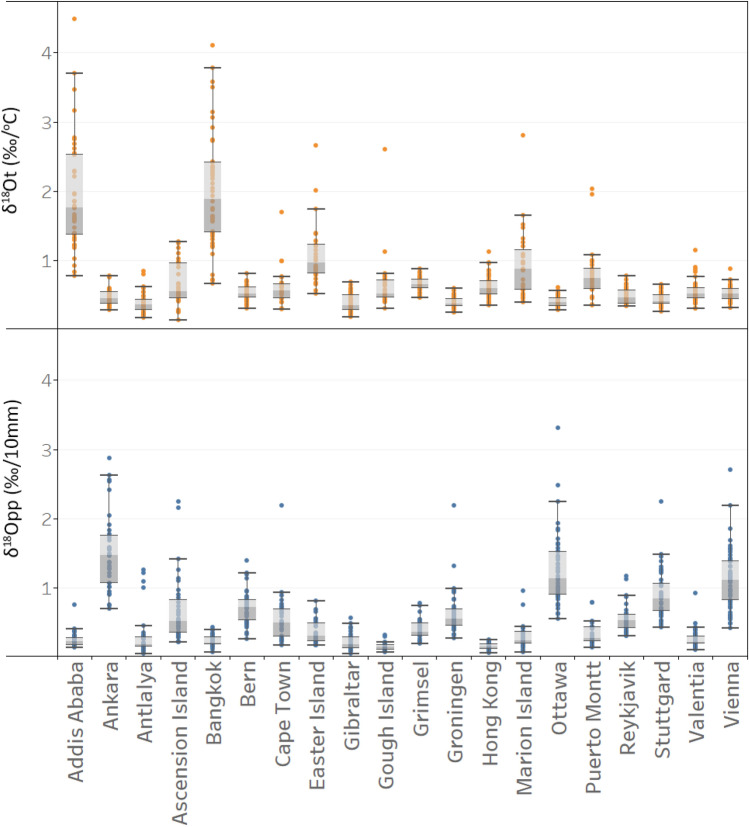
*δ*^18^O-dependency on T (*δ*^18^O_t_) and P (*δ*^18^O_pp_).

Addis Ababa and Bangkok showed the strongest *δ*^18^O dependency on T (*δ*^18^O_t_), with average *δ*^18^O_t_ dependencies of ~ 2 ‰/°C. For Ascension Island, Bern, Cape Town, Easter Island, Gough Island, Grimsel, Hong Kong, Marion Island, Puerto Montt, Valentia and Vienna, the *δ*^18^O_t_ dependency ranged from 0.5 to 1 ‰/°C, and for the rest of stations (Ankara, Antalya, Gibraltar, Groningen, Ottawa, Reykjavik and Stuttgart) the average *δ*^18^O_t_ was < 0.5 ‰/°C (Fig. 3). The average *δ*^18^O_t_ for oceanic island stations Ascension, Gough and Marion was highly variable for long-term data. The highest mean *δ*^18^O-dependency on P variation (*δ*^18^O_pp_) (> 1 ‰/10 mm) was found in stations from Group 1 (Ankara, Ottawa and Vienna) and the lowest (< 0.3 ‰/10 mm) was found in stations of Groups 2–4 (Addis Ababa, Bangkok, Gibraltar, Gough Island, Hong Kong, Marion Island and Valentia) (Fig. 3).

**Figure 4:**
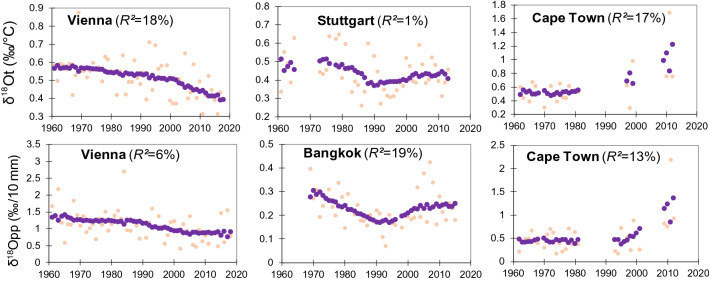
Nonparametric regression modelling (violet) of the annual time series of *δ*^18^O-dependency on T (*δ*^18^O_t_) and P (*δ*^18^O_pp_).

Significant decreasing long-term trends of *δ*^18^O_t_ (*R*^2^ = 18%) and *δ*^18^O_pp_ (*R*^2^ = 6%) were observed for Vienna where these values have decreased by a factor or 2 over the past 50 years (Fig. 4).

**Figure 5:**
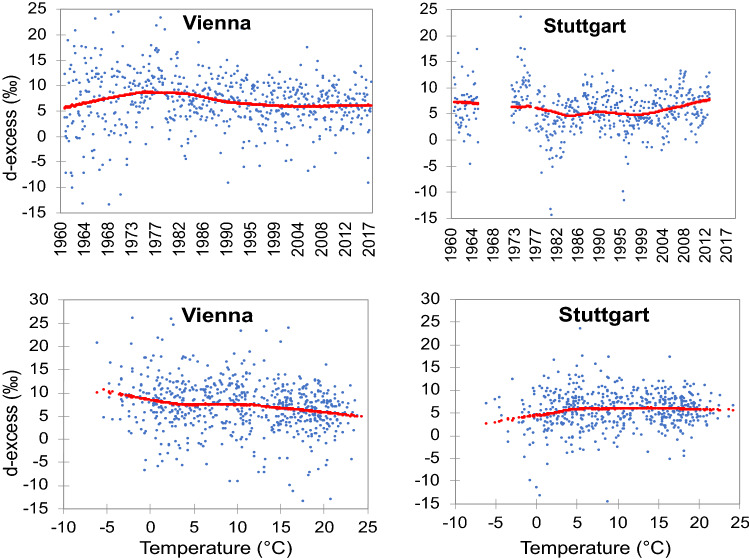
Nonparametric regression of *d*-excess variations over time and *d*-excess relationships with temperature for Vienna and Stuttgart (*R*^2^ was ~ 5% for each trend that was significant at *p* < 0.05).

Similarly, decreasing time trends of *δ*^18^O_t_ were found in Grimsel (*R*^2^ = 16%) (Figure SI-3). The decrease of *δ*^18^O_t_ trends in Vienna was correlated with a decrease in *d*-excess (Fig. 5)—a proxy for evaporation processes^30^. The negative correlation between *d*-excess and T in Vienna station suggests that sub-cloud evaporation has increased due to rising air temperatures over this decadal timeframe^19,30^ as confirmed by a downward *d*-excess shift since the 1980s (Fig. 5). Additionally, under the T growth the amount of P with lower isotopic composition and higher *d*-excess values decreased, and the share of warmer precipitation with more positive isotopic and lower *d*-excess values increased (Figure SI-4), as observed at other European sites^36,37^. The *δ*^18^O–T trend was lower at higher temperatures (Fig. 2) which explained the decrease of *δ*^18^O_t_ for Vienna and Grimsel (Fig. 4). Although less significant than *δ*^18^O_t_, the decrease of *δ*^18^O_pp_ for Vienna is characteristic of the increasing proportion of P originating from the recycled land moisture compared to the oceanic moisture sources.

Despite being in the same climatic zone as Vienna, Stuttgart exhibited diverse isotopic patterns related to T and *d*-excess (Figs. 4 and 5). A positive relation between *d*-excess and T for Stuttgart suggested a higher contribution of recycled moisture to precipitation (Fig. 5)^25,30^ and a higher impact of North Atlantic Oscillation (NAO) on the *δ*^18^O values, as seen by the U-shaped curve (Fig. 4) that corresponds closely to the NAO oscillation mode^19^.

In contrast to European stations, the *δ*^18^O_t_ and *δ*^18^O_pp_ in Cape Town rose over the past 30 years (Fig. 4). Simultaneous growth of the *δ*^18^O and T may be due to intensification of sub-cloud evaporation with rising T, along with a decrease of rainfall amount and a change of P frequency towards the more extreme values driven by the El Nino Southern Oscillation^38,39^. Non-linear time trends were found for *δ*^18^O_pp_ in Bangkok, with a negative trend until the 1990s and then rising in the > 2000s (Fig. 4) which coincides with modes of Pacific Decadal Oscillation and NAO^19^.

This study revealed the non-linearity between δ^18^O in precipitation and T, but also P over a 60-year period, which is a shorter interval than typically recorded in ice-cores. The non-linearity is explained by multiple external and local factors controlling the isotopic composition of precipitation including: (i) complex relationships related to recycled moisture contributions, (ii) large-scale convective processes and (iii) atmospheric-oceanic oscillations. While our findings make isotopic paleoclimate reconstructions and the study of large temperature shifts more challenging, it confirms that *δ*^18^O is a powerful integrative proxy of hydroclimatic variations that integrate more than local climate variations^19,40,41^. In keeping with previous studies^40,41^, *δ*^18^O in precipitation is generally well correlated with T in the mid- to high latitudes and with P in low latitudes, and this relationship holds if the predominant global and local atmospheric patterns remain stable. Accordingly, stable isotopes in precipitation and its proxies, but also their dependency on T and P, can be used to trace the ‘climate biases’ and reveals the instabilities caused by larger and smaller scale atmospheric processes that will require further study.

## Summary and perspectives

Analysis of long-term (60-year) global precipitation *δ*^18^O data and the relationships with P and T revealed diverse spatial climatic response patterns that cannot be simplistically unified in space and time. Nonparametric regressions showed T and P effects are be better explained by non-linear trends and hence can be used for more precise interpretations of climate dynamics. Moreover, over the multi-decadal sampling period we observed that the *δ*^18^O dependency on T and P was not constant over time, which indicates multiple effects of climate dynamics including global and regional scale hydrologic processes and oceanic cycles.

Nevertheless, despite the diversity of T and P responses on precipitation *δ*^18^O, some global stations could be considered as sentinel sites to better understand global circulation patterns and climate of the past and future. Particularly, stations located inland and at a large distance from the ocean where land mass influence has a stronger *δ*^18^O relationship to T and P, or differential *δ*^18^O–T relationship between warm and cold periods can be used to better calibrate reconstructed climate proxies, such as biotic (e.g., tree rings) and abiotic (e.g., ice and sediment cores) proxies. The strong *δ*^18^O–T relationship for Bern and Grimsel in the summer period indicates that these stations are well-suited to calibrate deciduous tree rings that integrate the isotopic signal of the summer evapotranspiration period. In contrast, the Ottawa region could be a reference area to better understand past climate through the interpretation of the isotope in the abiotic proxies, such sediment cores and groundwater, due to the strong relationship between the *δ*^18^O and T in the cold period under negligible evapotranspiration. However, even at stations with strong relationships between *δ*^18^O and T, none of these exhibited a strictly linear relationship due to the over-printing smaller-scale hydroclimate processes, such as transpiration and the contribution of the recycled moisture.

Stations in tropical or monsoonal-affected areas (e.g., Bangkok) highlight the influence of P effects on *δ*^18^O variability. However, low *δ*^18^O variability with P in these regions does not provide sufficient resolution for paleoclimate reconstructions, and hence these locations may be better suited as sentinels of long-term atmospheric and oceanic circulation patterns. Conversely, the strong *δ*^18^O signal with P associated with monsoonal activity in Bangkok and Hong Kong may be representative sites for the reconstruction of changes in large convective processes. However, detailed and higher frequency (e.g., event-based) observations of *δ*^18^O in precipitation in relation to the main drivers of monsoon and cyclonic activities such as larger scale atmospheric oscillations (Pacific Decadal Oscillation, Atlantic Multidecadal Oscillation, El Nino, El Nina, etc.) are needed to improve isotopic proxy interpretations. Similarly, stations with indistinguishable T and P effects and low *δ*^18^O variability revealed that other interdependent process are affecting T and the *δ*^18^O composition of meteoric water, rather than mainly local climatic conditions. Local T but also the P are thus indicators but not necessarily the cause of changes in hydrologic processes elsewhere. Based on two GNIP stations with long continuous *δ*^2^H and *δ*^18^O isotope records, we affirm that dual-isotope assays are crucial in going forward to better quantify the contribution of regional hydroclimate processes (i.e., evaporation dynamic, transpiration, recycled moisture and sub-cloud evaporation) to the isotopic composition in precipitation.

This study showed that the *δ*^18^O composition of global precipitation can be considered as an integrated hydroclimatic memory of many cumulative hydrological processes: sources of moisture and different pathways, different amounts of precipitation types and different durations of seasons. While 60 years of isotope records are remarkable, this is a relatively short period in terms of the geological and longer term climate impact dynamics, and accordingly the isotope analysis of global precipitation should be maintained or increased for assessing the natural or anthropogenic processes that contribute to past, present, and future hydroclimate variability.

## Methods

### Study area and data collection

Long-term records of stable isotopes and meteorological data for monthly composites of precipitation spanning ca. 60 years were obtained from the International Atomic Energy Agency (IAEA) the Global Network for Isotopes in Precipitation (GNIP)^42^. For long-term GNIP stations the data period covered 1960–2016 and with occasional station gaps at different times^19^. For each GNIP station, the available data for *δ*^18^O ranged from 280 (Puerto–Montt) to 667 (Vienna). Only a few stations had no gaps in their *δ*^18^O data series (Bangkok, Bern, Grimsel, Groningen, and Vienna) and small gaps existed for Hong Kong and Ottawa^19^.

We selected a subset of 20 GNIP stations spanning the entire isotope observation period from 1960-present (Fig. 1). These long-term stations covered inland and continental regions of Europe (Ankara, Antalya, Bern, Gibraltar, Groningen, Grimsel, Stuttgart and Vienna), Asia (Bangkok and Hong Kong), Africa (Addis Ababa and Cape Town), North America (Ottawa) and South America (Puerto Montt) as well as coastal and islands in Atlantic Ocean (Reykjavik, Valentia, Ascension and Gough Islands), Indian Ocean (Marion Island) and Pacific Ocean (Easter Island) Oceans^19,42^. Tableau Software v.2019.4.3 was used to generate the map on Fig. 1.

We used the precipitation-amount weighted monthly composite oxygen isotope (*δ*^18^O) data, monthly average surface air temperature and monthly precipitation amount for data interpretation. The isotope data for these selected GNIP stations were analysed over many decades using a wide variety of analytical techniques. For *δ*^18^O, most GNIP data before 2010 was measured by isotope-ratio mass spectrometry and CO_2_–H_2_O equilibration methods, and later on by laser spectroscopy. Prior to the 1990’s hydrogen isotope (*δ*^2^H) measurements were more difficult and resulted in many GNIP stations having only *δ*^18^O data with data gaps or altogether missing *δ*^2^H results. Therefore, we focused on the *δ*^18^O data time series for each station, knowing results for *δ*^2^H should be comparable through a meteoric water line relationship. Unfortunately, the lack of historical *δ*^2^H precluded assessment of long-term isotopic covariance for most stations (e.g., *d*-excess). The Vienna and Stuttgart stations had the longest time series for dual isotopes (*δ*^18^O, *δ*^2^H) with good analytical precision and few gaps in meteorological data for air temperature and precipitation amount. We determined *d*-excess value (*d*-excess = *δ*^2^H—8 · *δ*^18^O) for these stations to assess the influence of the regional hydroclimate processes on the isotope composition. In general, sub-cloud evaporation decreases and moisture recycling increases the *d*-excess^30^, hence *d*-excess could be used to better evaluate these processes.

### Data treatment

Data analyses were conducted using non-parametric regressions of the covariates (δ^18^O, P and T). We used local regressions, LOWESS smoothed cross-plots, and Kernel functions to weight the model outcomes^19,43^. Maximization of the coefficient of statistical explanation was evaluated by a significance metric, whereby the coefficient of determination (*R*^2^) ranged from 0% (no explanatory significance for *δ*^18^O and the regression model) to 100% where the covariate explained all the oxygen isotopic variance and the regression model^19,22^.

Grouping of GNIP stations by the relationship between the *δ*^18^O and meteorological parameters was based on the estimate *R*^2^, where the *R*^2^ threshold of the strong correlation was set at 30% of the explained isotope variability^22^.

Statistical analyses were conducted at the 95% confidence level (*p* < 0.05). We performed additional analysis of variance (ANOVA) and applied the F-test for double testing of the results. The ANOVA procedure assumes the regression is y (dependent variable) on x (independent variable).

The *δ*^18^O dependency on T (given per 1 ºC of air temperature) was calculated as following:

1 $$\delta^{{{18}}} {\text{O}}_{{\text{t}}} = \varDelta \delta^{{{18}}} {\text{O}}/\varDelta {\text{T}}$$

where *Δ δ*^18^O and *Δ*T are the difference between the monthly maximum and minimum *δ*^18^O values, and the recorded maximum and minimum monthly air temperature during the year, respectively.

The *δ*^18^O dependency on P (given per 10 mm of the precipitation) was calculated as following:

2 $$\delta^{{{18}}} {\text{O}}_{{{\text{pp}}}} = \varDelta \delta^{{{18}}} {\text{O}}/{1}0\cdot\varDelta {\text{PP}}$$

where *Δδ*^18^O and *Δ*PP are the difference between the monthly maximum and minimum *δ*^18^O values, and the recorded maximum and minimum precipitation amount during the year, respectively.

## Supplementary Information

Supplementary Information.
