# Supplementary material for: Temperature and precipitation effects on the isotopic composition of global precipitation reveal long-term climate dynamics
Source: Sci Rep. 2021 Sep 16;11:18503. doi: 10.1038/s41598-021-98094-6 (PMC8445972; doi:10.1038/s41598-021-98094-6)
Supplement: Supplementary file 1 — Supplementary Information. [file 41598_2021_98094_MOESM1_ESM.docx]

Supplementary information

**Temperature and precipitation effects on the isotopic composition of global precipitation reveal long-term climate dynamic**

Vystavna Y*., Matiatos I., Wassenaar L.I.

International Atomic Energy Agency, Isotope Hydrology Section, Vienna International Centre, Vienna, PO Box 100, Austria 1400.

**Table SI-1.** The coefficients of determination (*R^2^*) for nonparametric and linear regression between the *δ*^18^O and meteorological parameters (temperature and precipitation amount) at *p* < 0.05.

| Station | *Nonparametric regression* | | *Linear regression* | |
| --- | --- | --- | --- | --- |
|  | *δ*^18^O-temperature (*R^2^,* %) | *δ*^18^O-precipitation amount (*R^2^,* %) | *δ*^18^O-temperature (*R^2^,* %) | *δ*^18^O-precipitation amount (*R^2^,* %) |
| Stations with the strong *δ*^18^O-temperature relationship (*R^2^*>30%) | | | | |
| Ottawa | 70 | 2 | 68 | 3 |
| Grimsel | 64 | 6 | 63 | 7 |
| Vienna | 59 | 1 | 58 | n/s |
| Bern | 58 | 2 | 58 | 1 |
| Ankara | 54 | 11 | 55 | 8 |
| Stuttgart | 44 | n/s | 44 | n/s |
| Groningen | 39 | 2 | 39 | 2 |
| Stations with the strong *δ*^18^O-precipitation amount relationship (*R^2^*>30%) | | | | |
| Ascension Island | 15 | 35 | 12 | 31 |
| Bangkok | 7 | 44 | 1 | 39 |
| Stations with almost equivalent *δ*^18^O-temperature/precipitation amount relationships | | | | |
| Cape Town | 28 | 34 | 23 | 22 |
| Addis Ababa | 11 | 19 | 9 | 20 |
| Hong Kong | 49 | 46 | 43 | 38 |
| Puerto Montt | 31 | 21 | 31 | 20 |
| Valentia | 20 | 20 | 20 | 18 |
| Antalya | 19 | 13 | 20 | 9 |
| Stations with weak *δ*^18^O-temperature and *δ*^18^O-precipitation amount relationships | | | | |
| Easter Island | n/s | 6 | n/s | 8 |
| Gibraltar | 8 | 10 | 9 | 8 |
| Gough Island | 5 | 4 | 7 | 5 |
| Marion Island | 7 | 3 | 8 | 3 |
| Reykjavik | 12 | 6 | 11 | 4 |

n/s - means not significant


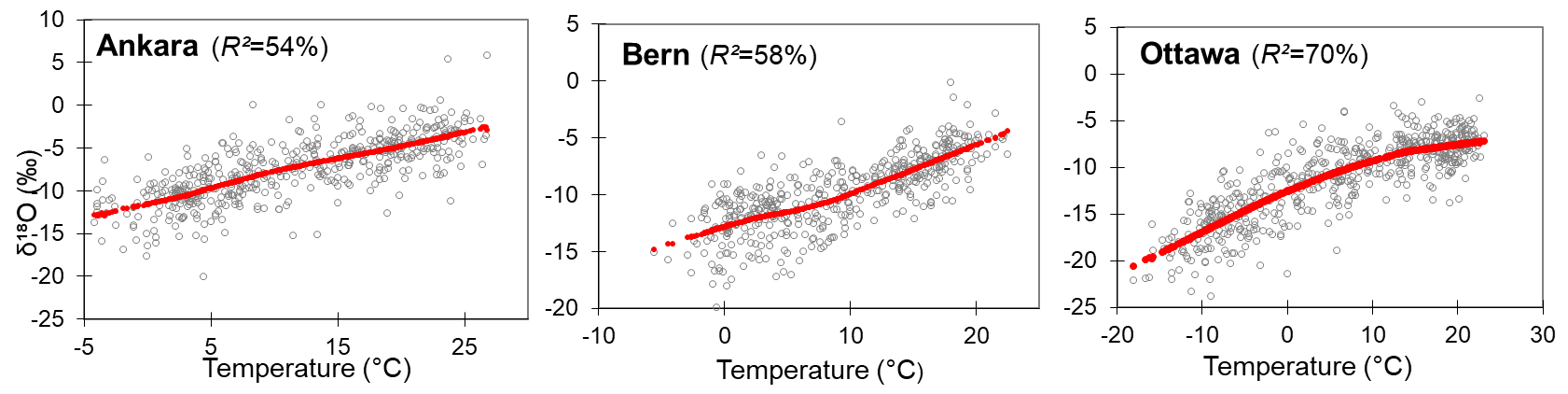


**Figure SI-1.** Polynomial trends fitted by nonparametric regression model for δ^18^O versus air temperature in selected long-term GNIP stations


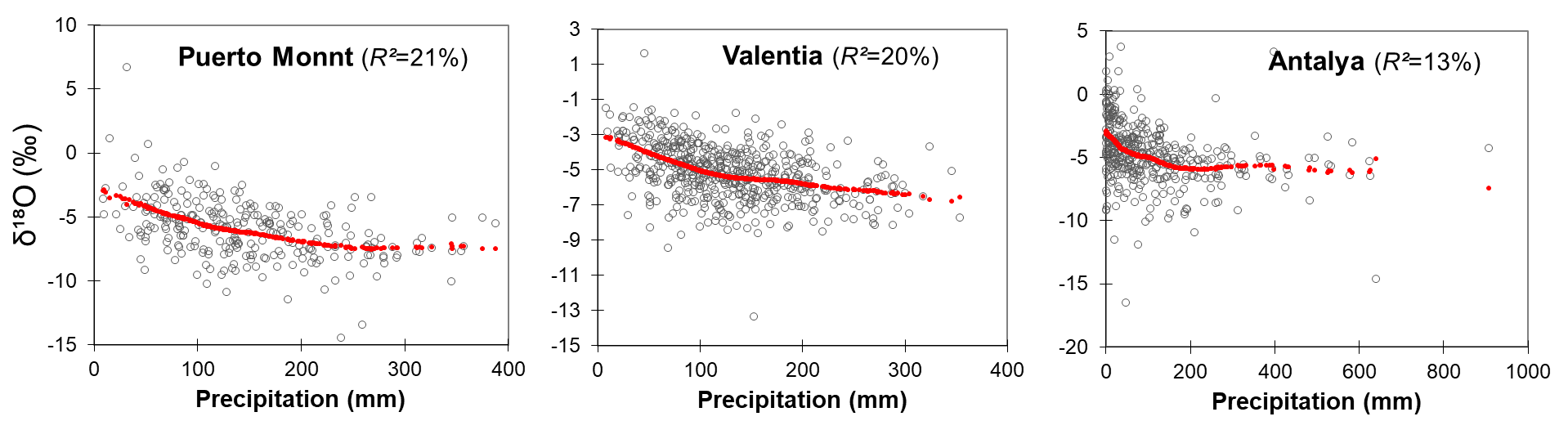


**Figure SI-2.** Polynomial trends fitted by nonparametric regression model for δ^18^O versus air precipitation amount in selected long-term GNIP stations


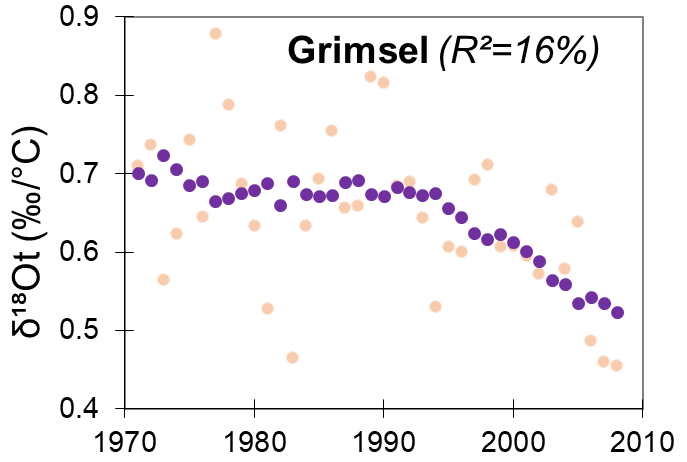


**Figure SI-3.** The nonparametric regression modelling (violet-coloured) of the annual time series of *δ*^18^O-dependency on T (*δ*¹⁸O_t_) and P (*δ*¹⁸O_pp_) variations.

a.


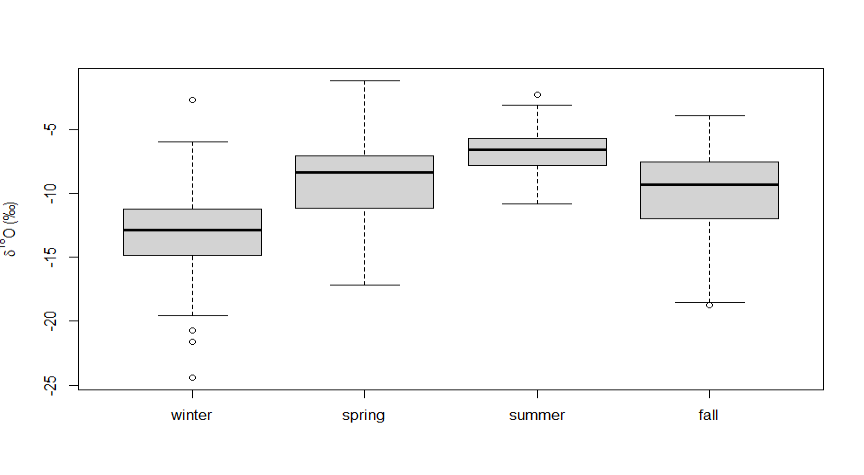


b.


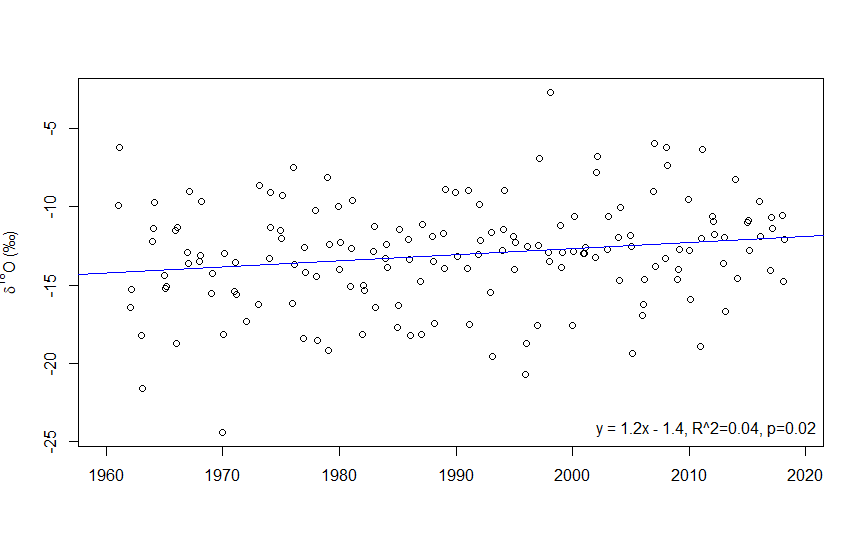


c.


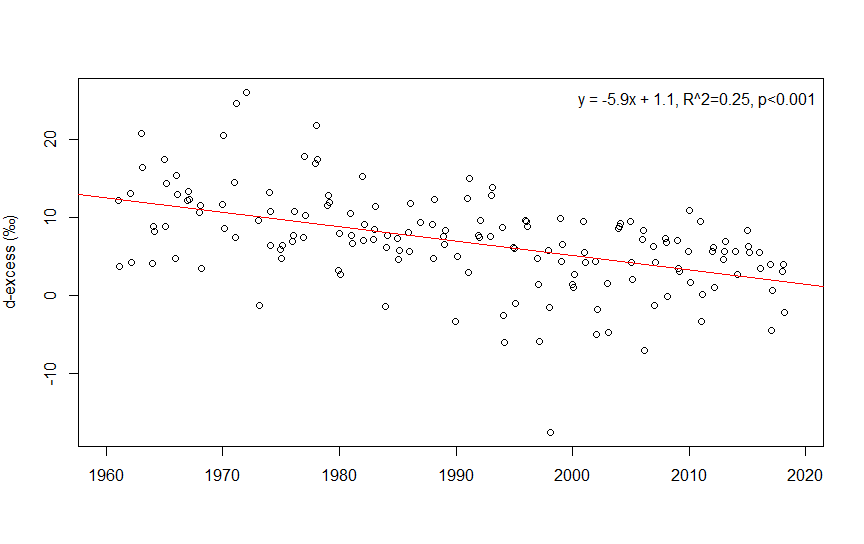


**Figure SI-4.** Long term isotopic composition in Vienna precipitation: (a) boxplots of the seasonality of *δ*^18^O; (b) *δ*^18^O value in winter precipitation (December – February) and (c) d-excess value in winter precipitation
